# Blended learning: Exploring nurse educators’ perspectives

**DOI:** 10.4102/hsag.v29i0.2659

**Published:** 2024-08-06

**Authors:** Daniel O. Ashipala, Emmanuel M. Sapalo, Peneyambeko I. Shikulo

**Affiliations:** 1 Department of General Nursing Sciences, Faculty of Health Sciences and Veterinary Medicine, University of Namibia, Rundu, Namibia; 2 Department of General Nursing Sciences, Faculty of Health Sciences and Veterinary Medicine, University of Namibia, Oshakati

**Keywords:** blended learning, perspectives, utilisation, nurse educators, teaching method

## Abstract

**Background:**

In a rapidly evolving educational landscape, blended learning is becoming an increasingly popular transition from traditional forms of learning and teaching to e-learning. It is therefore important that lecturers adapt their practice and transform their teaching in line with the online platform in use, as this has the potential to benefit students, lecturers and the institution alike. However, little research exists regarding the perspectives of nurse educators on the use of blended learning as a teaching method.

**Aim:**

The study aimed to explore and describe the perspectives of nurse educators on the use of blended learning as a teaching method at the Faculty of Health Sciences at a university in Namibia.

**Setting:**

The study was conducted at a public nurse education institution in Namibia.

**Methods:**

A qualitative exploratory, descriptive design that was contextual was applied to collect data from a convenient sample of 15 lecturers using semi-structured interviews.

**Results:**

Four themes emerged in this study, namely, understanding of blended learning, benefits of utilising blended learning, challenges of utilising blended learning, and recommendations to ensure effective use of blended learning.

**Conclusion:**

The study findings identified potential areas of both strengths and shortcomings in nurse educators’ use of blended learning as a teaching and learning strategy.

**Contribution:**

These findings may be used to develop ongoing strategies and targeted interventions that can strengthen nurse educators’ abilities to design learning environments that are conducive to blended learning.

## Introduction

Blended learning is one of the most significant forms of learning in the 21st century, because it takes advantage of both online and face-to-face learning (Capone, De Caterina & Mazza 2017). The basic principles of blended learning were first applied in corporate and higher education in the 1960s, but the term was first used in 1999, when the American Interactive Learning Center began to release software programs designed for teaching over the internet (Lakhal & Meyer 2020). Blended learning, a pedagogical approach that combines traditional face-to-face instruction with online learning activities, emerged as a response to the growing integration of technology in education worldwide. The roots of blended learning can be traced back to the early 2000s, when advancements in internet technology paved the way for the development of online learning platforms and tools. Higher education institutions and educators subsequently began to recognise the potential of blending online resources with traditional classroom instruction to enhance learning outcomes and cater to diverse student needs (Bonk & Graham 2012).

In Africa, more people started using blended learning from the late 2000s (Adeniyi et al. 2024). This was influenced by various factors; for example, city-dwellers in Africa were increasingly using the internet and digital devices. Other factors included the need for learning options for older people and working professionals, and the need to address problems such as a poor education system and a lack of education facilities (Singh, Steele & Singh 2021). The implementation of blended learning in Africa has been marked by innovative approaches tailored to local contexts and needs. These include partnerships between educational institutions and technology providers to develop customised platforms and content, as well as initiatives to train educators (Castro 2019). Some African countries have advanced faster than others, driven by a collective effort to harness the potential of technology to democratise access to education and improve learning outcomes across the continent (Rambe & Moeti 2017).

Blended learning has also emerged as a new teaching and learning method in undergraduate nursing and midwifery education (Leidl, Ritchie & Moslemi 2020). While there is a great deal of literature on nursing students’ experiences on the use of blended learning, no studies were found investigating nurse educators’ experiences.

The University of Namibia (UNAM) embarked on its journey to blended learning in 2013, marking a significant shift in its approach to higher education delivery. Recognising the potential of blending traditional face-to-face instruction with online learning components, UNAM sought to enhance accessibility, flexibility and quality in its educational offerings (UNAM 2022). The adoption of blended learning at UNAM was driven by a vision to leverage technology to overcome geographical barriers, cater to diverse student needs and promote lifelong learning opportunities.

The implementation of blended learning at UNAM followed a strategic and phased approach, integrating digital tools and platforms into existing curricula while providing support and training for faculty members. Initially, pilot projects were launched in selected departments to test the feasibility and effectiveness of the blended learning models in the Namibian context (Magesa & Josua 2022). These projects involved the development of online modules, multimedia resources and interactive activities, complemented with face-to-face sessions to facilitate engagement and collaboration among students.

Over the years, evidence has emerged regarding the effectiveness of blended learning at UNAM, with studies showing improvements in student engagement, satisfaction and academic performance. These were attributed to the personalised learning experiences and flexibility afforded by the blended approach (UNAM 2022). Moreover, blended learning has enabled UNAM to reach a wider audience, including working professionals and learners in remote areas, thereby increasing access to higher education and fostering inclusive learning environments. As UNAM continues to refine its blended learning initiatives, ongoing evaluation and feedback mechanisms are essential to ensure continuous improvement and alignment with the evolving needs of students and stakeholders. By harnessing the potential of blended learning, UNAM remains committed to advancing its mission of providing quality education that empowers individuals and contributes to national development goals in Namibia.

**FIGURE 1:**
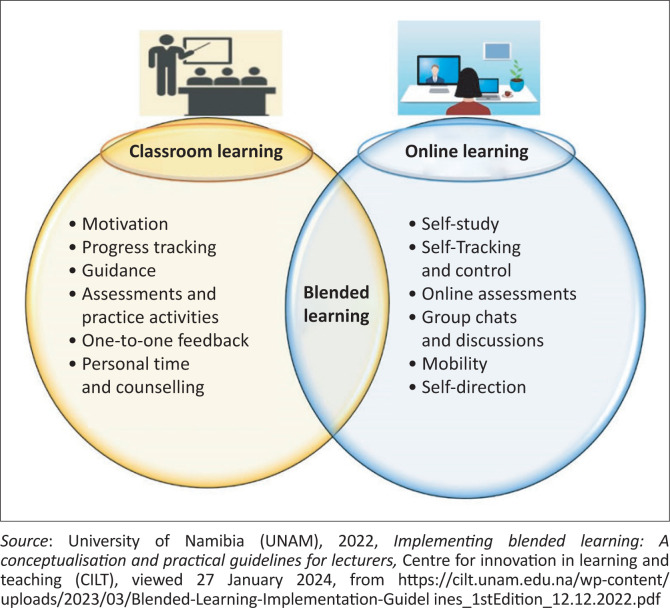
Blended learning (designing instructions for e-learning).

Figure 1 shows how blended learning is being utilised at UNAM in the online drivermodel or face-to-face approach.

Flexibility is another major advantage of e-learning, as it enables learners to take classes anywhere, anytime (Al Rawashdeh et al. 2021). This model involves the nurse educator actively presenting core content and facilitating access to supplementary materials for further study (Vaughan, Cleveland-Innes & Garrison 2013). During this phase, assessment is made easier as the assessments in this model offer lecturers the opportunity to establish a more personal connection with their students, fostering a supportive learning environment. This interpersonal interaction allows for better discussions, individualised feedback, and the identification of students’ strengths and areas that may require additional attention (Karimi Moonaghi & Mohsenizadeh 2019).

Karimi Moonaghi and Mohsenizadeh (2019) agreed that applying the online driver model/face-to-face approach helps nurse educators see if students understand the material by watching how they participate in class and react to activities. This helps them change how they teach right away, thus addressing any misunderstandings or things that students do not understand immediately. On the other hand, students are instructed to complete most of the curriculum online through self-learning and contact the lecturer either on a need basis or mandatorily, as instructed by the programme (Chaudhuri 2023). This helps lecturers reduce pressure on physical resources and enhance communication, as well as assisting them to manage larger class groups more easily (Pottle 2019). In addition, since the institution has started using blended learning, nurse educators have conducted formative assessments online, while summative assessments take place face-to-face. Some educators have chosen to split the formative assessment, with some taking place in a physical venue and others online (UNAM 2023). Therefore, this study was conducted to explore and describe the perspectives of nurse educators on the use of blended learning as a teaching method at the Faculty of Health Sciences at a university in Namibia.

## Research methods and design

### Study design

A qualitative exploratory, descriptive design that was contextual was applied to explore and describe the perspectives of nurse educators on the use of blended learning as a teaching and learning strategy at UNAM (Polit & Beck 2020). A qualitative approach was applied because it can assist in the investigation of themes such as life experiences (Emami et al. 2012). Moreover, according to Maree (2016), a qualitative research design is naturalistic, that is, it focuses on the natural settings in which interactions occur. In light of this, it was an appropriate design for evaluating nurse educators’ perspectives on blended learning. A qualitative approach is used to explore how people make sense of their surroundings, and their experiences and understanding of a phenomenon. The qualitative exploratory and contextual design thus allowed for an in-depth exploration and understanding of nurse educators’ perspectives of utilising blended learning as a teaching method at the Faculty of Health Sciences in Namibia.

### Study setting

The study was conducted at a satellite campus of UNAM situated in the northeast of Namibia. Academic programmes offered include management sciences, economics, education and nursing sciences, including a 4-year, full-time undergraduate Bachelor of Nursing Science (clinical) Honours programme. All the nursing courses in this programme were provided face-to-face prior to the coronavirus disease 2019 (COVID-19) pandemic. In 2013, the UNAM started the transition to blended learning, with the aim of improving availability, effectiveness and reliability (UNAM 2022). This embrace of blended learning was driven by technological innovation to bridge distances, meet students’ needs and inspire continuous learning.

### Population and sampling

The targeted population and sample size were 15 nurse educators at the School of Nursing and Public Health. This population group was targeted because it used blended learning prior to the pandemic and was available (Mweshi & Sakyi 2020). This was seen as a suitable sampling technique as the intention of qualitative research is not to generalise findings (Brink, Van Der Walt & Van Rensburg 2018). The inclusion criteria were lecturers working as nurse educators in the School of Nursing and Public Health, UNAM, who were willing to participate in a face-to-face interview by signing the consent form. The sample of 15 nurse educators was considered rich and adequate as no new insights were emerging (Brink et al. 2018). Lowe et al. (2018) and Gray, Grove and Sutherland (2017) indicated that data saturation occurs when maximum information has been obtained.

### Data collection

Data for this study were collected during September 2023 using semi-structured, face-to-face interviews of approximately 45 min each. This allowed the researchers to gather information from the nurse educators on their lived experiences within their own contexts (Lawrence et al. 2022). The interviews included open-ended and follow-up questions, which enabled the nurse educators to freely share their perspectives (Adeoye-Olatunde & Olenik 2021). An interview guide was drawn up based on the research questions, the study objectives and a literature review. The central question posed was: What are your perspectives of utilising blended learning as a teaching method at the Faculty of Health Sciences? Nurse educators were approached by the researcher, who explained the aim of the study, after which those who agreed to take part were asked to sign a consent form. A pilot study was conducted with two nurse educators from another satellite campus of UNAM to test the interview guide and its suitability for generating relevant data. This demonstrated that the questions were suitable. Data generated from the pilot interviews were not included in the main study.

### Data analysis

The researchers in this study employed thematic analysis to analyse the data, because it enabled them to arrange the data into themes and subthemes. This approach is considered to be the most suitable in qualitative data analysis (Polit & Beck 2017). The researcher repeatedly read the interview transcripts to gain insight, before creating preliminary data codes that helped to identify themes and groupings within the data. These diverse codes were then narrowed down into recurring themes and categories. Prior to writing up the research findings, main themes and subthemes were decided upon and named. Following that, meaning units were assigned to the major and subcategories. The researcher and the impartial co-coder then highlighted the meaning units associated with the identified categories. It was requested that the impartial coder, separate from the researcher, also analyse the data using thematic analysis. The two analyses were then contrasted to ensure they were reliable. Table 2 displays the themes and subthemes.

### Measures to ensure trustworthiness

Trustworthiness was ensured by using Lincoln and Guba’s (1985) model, which ensures the credibility, dependability, confirmability and transferability of a study (Polit & Beck 2020). Credibility was realised by holding pilot interviews, establishing data saturation, lengthy participant engagement (8 weeks), active participation, observing and interacting with nurse educators, recording interviews, member checking (by replaying the recordings to the nurse educators) and validating the transcripts with the research supervisor. Transferability was achieved by incorporating rich descriptions of the perspectives of the nurse educators, as well as their contexts, so that their meaning was evident to outsiders (Korstjens & Moser 2018). Dependability was ascertained via a peer debriefing with researchers not involved in the study, as well as extended engagement with the interviewees and member checking. An inquiry audit using an external reviewer ensured confirmability. Finally, reflexivity was established by the researcher remaining aware of his roles in the study and his ongoing reflection on his personal behaviours and how these might affect the research (Polit & Beck 2020). This reflection was made possible through the use of research diaries, which the author incorporated into his field notes.

### Ethical considerations

Permission to conduct the study was obtained from the UNAM ethics committee (SoNEC40/2023) and the Ministry of Health and Social Services (Ref:22/3/1/2). Participation was voluntary and informed consent was obtained from all interviewees. Complete confidentiality was not guaranteed because the researchers could not control what the educators would talk about after the interviews. All nurse educators were given a chance to participate. To the best of the researchers’ knowledge, no harm was done to any participant and no names are divulged or linked to any data. All audio recordings, interview transcripts and field notes are stored in a computer protected by a password known only to the researcher. The gathered data will remain confidential and available only to the researcher, the research supervisor and the independent coder. Data will be disposed of after five years in accordance with the university’s policy.

## Results

### Demographic characteristics of nurse educators

The participants consisted of 11 females and 4 males. Eleven of the educators were lecturers and four were senior lecturers. The characteristics of the study nurse educators are given in Table 1.

**TABLE 1:** Demographic characteristics of the nurse educators.

Participant	Gender	Age (years)	Years of experience	Rank/position
P1	Male	56	15	Senior lecturer
P2	Female	63	9	Lecturer
P3	Female	33	13	Senior lecturer
P4	Male	38	5	Senior lecturer
P5	Female	54	9	Lecturer
P6	Female	45	14	Lecturer
P7	Female	48	16	Lecturer
P8	Male	42	9	Lecturer
P9	Female	44	11	Lecturer
P10	Female	56	10	Lecturer
P11	Male	51	14	Lecturer
P12	Female	38	10	Lecturer
P13	Female	41	13	Lecturer
P14	Female	63	9	Lecturer
P15	Female	54	11	Lecturer

### Themes and subthemes

From the data analysis, nine subthemes emerged, which were clustered into four major themes, namely, nurse educators’ understanding of blended learning, the benefits of utilising blended learning, the challenges of utilising blended learning, and the recommendations to ensure the effective use of blended learning. The four themes that emerged from the data analysis are indicated in Table 2.

**TABLE 2:** Themes and subthemes that emerged from data analysis.

Themes		Subthemes
1.	Understanding of blended learning	1.1. Integration of face-to-face and online learning
2.	Benefits of utilising blended learning	2.1. Allows educators the chance to focus on own studies and personal interests 2.2. Fosters self-directed learning 2.3. Assessment and evaluation of tests made easier
3.	Challenges of utilising blended learning	3.1. Limited student participation in online sessions 3.2. Poor resources and internet connectivity 3.3. Cheating in online tests
4.	Recommendations to ensure effective use of blended learning	4.1. Continuous training for educators on how to utilise online platforms and equipment 4.2. Improvement of internet quality on campus

#### Theme 1: Understanding of blended learning

This theme is a description of the nurse educators’ understanding of the term blended learning obtained from a thematic analysis of the data that were collected. In this theme, nurse educators highlighted the integration of face-to-face and online learning.

**Subtheme 1.1: Integration of face-to-face and online learning:** Nurse educators in this study expressed their understanding of the term *blended learning* as the integration of face-to-face and online learning:

‘Blended learning for me, it’s a way in which we mixing learning online and learning face-to-face. And we’re doing this in an integrated way.’ (P1)

‘In short, that’s when you incorporate face-to-face with online or technological teaching, and learning, so it’s a combination of face-to-face, and online learning.’ (P4)

‘Blended learning means educating students through electronic means, in combination with the traditional way of teaching which is a face-to-face teaching practice.’ (P2)

#### Theme 2: Benefits of utilising blended learning

This theme entails a description of the nurse educators’ responses when they were asked to share their perspectives on the benefits of using blended learning as a teaching method. Based on the data collected, the following subthemes were generated: allows educators the chance to focus on own studies and personal interests; fosters self-directed learning; and assessment and evaluation of tests made easier.

**Subtheme 2.1: Allows educators the chance to focus on own studies and personal interests:** According to the interviewees, blended learning provides flexibility and the freedom to focus on personal studies, as well as time to travel and participate in different community activities:

‘The only benefit that I’ve seen through the use of blended learning is that you have an opportunity to do other things. For example, research, because you can preload your lecture online in the students can access it.’ (P2)

‘[*A*] benefit of blended learning is that it allows it frees time for both the students and the lecturer and gives freedom in terms of learning.’ (P1)

**Subtheme 2.2: Fosters self-directed learning:** The nurse educators revealed that blended learning enables self-directed learning for students:

‘With blended learning, it was easier, for one example, to learn during their own time, you will be able to access different activities and different teaching materials or learning materials at your convenient time when you feel ready to interact with that material.’ (P4)

‘Even the live classes can be recorded [*for*] those students who are absent or who are unable to make it.’ (P5)

**Subtheme 2.3: Assessment and evaluation of tests made easier:** Nurse educators commented that the marking of test papers and other assessments is a factor that contributes to an excessive workload. Blended learning has certain benefits in this regard as the use of online assessment means that assessments and tests are evaluated faster and more easily.

‘We would also see that when it comes to formative evaluation of students, you can do it easily because your workload is reduced since the marking is done online.’ (P2)

‘You don’t take time on assessment, marking an assignment or marking assessment that you have given students.’ (P7)

They added that online platforms like Moodle make things easier, as most marking is done automatically.

‘… the marking can be done automatically and then for you is just to record the marks.’ (P5)

#### Theme 3: Challenges of utilising blended learning

This theme reflects on the drawbacks that nurse educators experienced in utilising blended learning as a teaching method at UNAM. The following subthemes emerged from the data analysis: limited student participation in online sessions; poor resources and internet connectivity; and cheating in online tests.

**Subtheme 3.1: Limited student participation in online sessions:** The educators mentioned that despite appreciating the flexibility, there is limited and poor participation and engagement of students in online classes because of a lack of motivation and technical difficulties:

‘You will find that some students will not attend and only later they tell you they were not allowed into the classroom because it said maximum nurse educators reached.’ (P5)

‘The difficulty that I saw was student attendance; it is very difficult to motivate students to participate.’ (P2)

**Subtheme 3.2: Poor resources and internet connectivity:** Nurse educators reported that for online lessons to take place, there is a need for good internet connectivity and proper equipment for online classes, which, as a number of nurse educators in this study stated, is lacking, thus hindering them from offering effective classes online:

‘My main experience was the challenges of network sometimes, and sometimes the system can fail you. You schedule a lesson for students and the system is off, or along the line the system just it kicks you off.’ (P13)

‘During the time of assessment or teaching not everyone has the same access to the internet.’ (P12)

‘Some students who stay in shacks in they will not be able to connect because there is no electricity within their homes.’ (P2)

**Subtheme 3.3: Cheating in online tests:** Nurse educators reported that cheating by students in online tests was a big concern when using blended learning, and the evaluation of students is very challenging.

‘With regard to cheating, I think to the assessment most of the students would be seen not very serious with the content, because most of the time it’s an open book test. And they have to do it in groups.’ (P4)

‘When it comes to students’ evaluations, you see that when you use the online mode, students can get very high marks, but when you give the test face-to-face, students get very low marks.’ (P8)

#### Theme 4: Recommendations to ensure effective use of blended learning

This theme presents suggestions by different nurse educators on how to promote the effective use of blended learning at the UNAM. The subthemes are continuous training for educators on how to utilise online platforms and equipment, and improvement of internet quality on campus.

**Subtheme 4.1: Continuous training for educators on how to utilise online platforms and equipment:** Nurse educators mentioned that there is a need for continuous training, because most of them are used to the traditional way of teaching, that is, face-to-face; when it comes to online teaching and the use of electronic devices, they are faced with some huge challenges:

‘There is also need for the university to continuously train lecturers on blended learning, especially the use of electronic devices.’ (P11)

‘As educators we need to be more equipped with technological advancement skills so that blended learning should be encouraged more, because when I look at it now we have really gone back to the traditional way of teaching, but blended learning can still be utilised.’ (P5)

‘I think continuous training or continuous on-the-job training of the lecturers.’ (P10)

**Subtheme 4.2: Improvement of internet quality on campus:** University of Namibia had a contract with Telecom Namibia, which was the first company to provide internet to all students, giving them pocket Wi-Fi and data-loaded subscriber identity module (SIM) cards. However, because of poor internet connections, the agreement was terminated. Later, a new agreement with Mobile Telecommunications Limited (MTC) was proposed, which was thought to offer better services; however, the educators mentioned that there is still a need for improvement in the quality of the internet being provided:

‘I think the university must be negotiated with internet providers to boost connectivity, especially within the campus.’ (P9)

‘… maybe the 5G should be available at the campus … if the majority of the students are there they will not be able to access it; the internet comes down and up and off and on, which does not support online learning.’ (P7)

## Discussion

Some of the nurse educators mentioned that a blended method entails the combination of traditional teaching and online learning. This is in line with a study conducted by Hrastinski (2019), which defined blended learning as a combination of traditional classroom methods of teaching and online modalities. In addition, Wright (2017) described blended learning as a combination of face-to-face traditional learning and online learning methods. Similar findings were reported by Dakhi, Jama and Irfan (2020), that is, that blended learning is the integration of face-to-face and online instruction.

The nurse educators revealed that blended learning enables self-directed learning for students as it allows them to take responsibility for their own learning in their own time. These findings are supported by Bosch (2017) and Adinda and Mohib (2020), who found that blended learning environments help students to develop their self-directed learning skills.

The nurse educators also stated that the use of online platforms such as Moodle for marking is effective and does most of it automatically. Similar findings emerged from a study by Baleni (2015), that is, it provides immediate feedback to students. The study further showed that e-assessment tools are used progressively; lecturers benefit in terms of both faster marking time and fewer administrative costs, while for students, online quizzes give prompt and comprehensive feedback and, importantly, enhance flexibility regarding the time and place for taking the assessment task. Dangwal (2017) also found that online assessment offers immediate feedback and helps to make the evaluation system more formative, transparent and faster.

Nurse educators mentioned that despite appreciating the flexibility, there is limited or poor participation and engagement of students in online classes because of a lack of motivation and technical difficulties. These findings are in line with a study by Wahid, Afni and Suarni (2022), which found that students feel less motivated when attendance is not compulsory. Similar concerns were raised in a study conducted by Fazza and Mahgoub (2021). In addition, for online lessons, there needs to be good internet connectivity and proper equipment, including electricity. These findings are similar to those of Wahid et al. (2022) and Mallillin et al. (2020), who emphasised that most problems in online teaching relate to internet connectivity and hardware.

In addition, cheating in online tests is a big concern, which is as per a study by Kocdar et al. (2018). Some of the types of cheating include impersonation, looking at others’ answers and ghost writing. In addition, Yazdi and Hatami (2023) and Tomas, Munangatire and Iihuhua (2022) raised the issue of academic dishonesty in formative assessments that are conducted online, as this is an unprotected environment.

Nurse educators stated that there is a need for ongoing training in the use of online platforms and equipment, because most of them are used to teaching face-to-face. Similar findings were reported by Roszak and Kołodziejczak (2017), who indicated that the improvement of teachers’ skills and ICT competencies is a necessity and should be ongoing and regular. Additionally, Burns (2020) indicated that educators have to invest considerable time upfront to learn how to create online learning material, that is, they are required to develop new skills.

In a study by Ynn and Poon (2021) on the perceptions of blended learning among three-dimensional animation lecturers at a higher education institution, similar findings regarding the need for improved internet quality were obtained. This indicates that technological concerns are perceived as the biggest obstacles to blended learning and that the availability of high-speed internet is critical. In a study conducted by Mallillin et al. (2020), nurse educators noted that a good internet connection or Wi-Fi could improve certain issues in online classes; thus, Wi-Fi providers should consider this as a part of their services in the community. Similarly, Dangwal’s (2017) study indicated that blended learning should proceed according to a well-planned design that includes all individuals along the educational hierarchy.

### Limitations

This study focused on nurse educators at a single university; thus, the findings cannot be generalised. This limits the transferability of the findings to other contexts.

### Recommendations

The nurse educators in this study strongly recommended the introduction of an online training programme for nurse educators. It is also a good idea to try different learning models in a blended classroom, such as the flipped classroom, which involves taking the traditional roles in a classroom. Another way to make a blended classroom more successful is to use videos, for example, asking students to give a video presentation of their understanding of the course material as an assignment.

Firstly, it is important to keep up to date with new tools and trends in teaching in order to stay current. Strong and reliable internet connectivity is also key. By making sure that nurse educators keep learning and improving, and that the internet works well, schools can create an effective learning environment, which will help both educators and students. Further research should be conducted on the utilisation of blended learning among various faculties and on other campuses using a quantitative design or a comparative approach.

## Conclusion

The purpose of this study was to explore nurse educators’ perspectives on the use of blended learning as a teaching method at UNAM, with a view to understanding the challenges they face. The research revealed that there are a number of challenges that must be overcome before blended learning can be a success, including limited student participation in online sessions, inadequate resources and internet connectivity, and instances of cheating in online assessments. Additionally, there is a need for ongoing training for educators in the utilisation of online equipment. These findings can be used to develop ongoing strategies and targeted interventions that can strengthen nurse educators’ ability to design learning environments that are conducive to blended learning.
